# Pilot and feasibility studies: extending the conceptual framework

**DOI:** 10.1186/s40814-023-01233-1

**Published:** 2023-02-09

**Authors:** Christine Bond, Gillian A. Lancaster, Mike Campbell, Claire Chan, Saskia Eddy, Sally Hopewell, Katie Mellor, Lehana Thabane, Sandra Eldridge

**Affiliations:** 1 Primary Care, Institute of Applied Health Sciences, University of Aberdeen, Aberdeen, UK; 2 Keele Clinical Trials Unit, School of Medicine, Keele University, Keele, UK; 3 School of Health and Related Research, University of Sheffield, Sheffield, UK; 4 Barts and the London Pragmatic Clinical Trials Unit Centre for Evaluation and Methods Wolfson Institute of Population Health, Queen Mary University of London, London, UK; 5 Centre for Evaluation & Methods, Wolfson Institute of Population Health, Queen Mary University of London, London, UK; 6 Oxford Clinical Trials Research Unit/Centre for Statistics in Medicine, Nuffield Department of Orthopaedics, Rheumatology and Musculoskeletal Sciences, University of Oxford, Oxford, UK; 7 Health Research Methods, Evidence, and Impact, McMaster University, Hamilton, ON Canada; 8 St Joseph’s Healthcare—Hamilton, Hamilton, ON Canada; 9 Faculty of Health Sciences, University of Johannesburg, Johannesburg, South Africa

**Keywords:** Internal pilot, External pilot, Definitions, Concepts, Uncertainties, Randomised controlled trial (RCT), Feasibility, Reporting, Framework

## Abstract

In 2016, we published a conceptual framework outlining the conclusions of our work in defining pilot and feasibility studies. Since then, the CONSORT extension to randomised pilot and feasibility trials has been published and there have been further developments in the pilot study landscape. In this paper, we revisit and extend our framework to incorporate the various feasibility pathways open to researchers, which include internal pilot studies. We consider, with examples, when different approaches to feasibility and pilot studies are more effective and efficient, taking into account the pragmatic decisions that may need to be made. The ethical issues involved in pilot studies are discussed. We end with a consideration of the funders’ perspective in making difficult resource decisions to include feasibility work and the policy implications of these; throughout, we provide examples of the uncertainties and compromises that researchers have to navigate to make progress in the most efficient way.

## Introduction

In 2016, we published a conceptual framework for defining feasibility and pilot studies in preparation for a randomised controlled trial [1].The paper has been extensively cited and our definitions have been widely accepted by funding bodies such as the UK NIHR (National Institute for Health Research) [2] and the HRB (Health Research Board) in Ireland [3]. However, there have also been further developments in the pilot study landscape. In this article, we present a rationale for extending the conceptual framework to incorporate these developments and discuss their implications for funders, policy makers and researchers.

The 2016 conceptual framework, built on work by members of our group [4, 5], other researchers [6] and the guidance from the National Institute for Health Research (NIHR) [7] and Medical Research Council (MRC) [8] funding bodies. It is summarised in the first section of this article. The original reason for developing the framework was multifactorial. One key driver was a realisation that the terms ‘pilot’ and ‘feasibility’ study were used inconsistently and interchangeably.

Scientific communication should ensure as far as possible that words are used with a common understanding of their meaning. It is also important for the scientific community that research findings are disseminated so that the results can be used by others. We initially developed definitions for the terms pilot and feasibility [1] in line with the then NIHR [7] and MRC [8] recommendations and other current informed opinion (Table 1). We found that many publications labelled as pilot studies appeared to have these labels because they had limitations, rather than because they were being conducted as a true pilot for a subsequent definitive study. Examples of limitations include a small sample size, lack of a power calculation (so they were not definitive trials), lack of meaningful clinical outcomes or short-term follow-up. We also found that some authors incorrectly considered the use of surrogate outcomes as pilot work [9]. In effect, many studies labelled as pilot or feasibility were neither definitive nor undertaken to inform subsequent research. We suggested that the terms ‘pilot’ and ‘feasibility’ had historically been misappropriated and the labels devalued. This potentially explained why, at the time of publishing our conceptual framework in 2016, journal editors appeared to be unwilling to publish pilot and feasibility studies (see Table 1), thus limiting their availability and exposure to other researchers in the field.

**Table 1:** A chronology of guidance and evidence illustrating the evolving feasibility and pilot study landscape from our perspective

Time frame	Date and author	Key points
Pre 2008	2004 Lancaster [4]	Seminal publication: No formal guidance on what constitutes a pilot study; recommendations made for good practice. Editors reluctant to publish.
2008-15	2008 MRC Guidance [8]	No absolute definition for pilot or feasibility studies but emphasised the need to conduct pilot and feasibility work to identify and address problems that might occur in subsequent RCTs
	2010 Thabane [5]	Demonstrated inconsistent and synonymous use of terms pilot and feasibility
	2010 NIHR [7]	Stated that the terms feasibility and pilot were mutually exclusive
	2010 Arain [6]	Demonstrated that in the literature studies described as feasibility or pilot had different characteristics. Editors *‘loathe to publish studies described as 'pilot”.*
	2011	Workshop on pilot studies, led by Sandra Eldridge (SE), Gillian Lancaster (GL), Mike Campbell (MC) and Sally Kerry, and attended by Christine Bond (CB), held at Annual Scientific meeting of the Society of Academic Primary Care in Bristol [10]. Outcome of the workshop was a decision to develop a CONSORT extension for pilot and feasibility studies.
	2012	The Pilot and Feasibility Studies (PAFS) group was formed by SE, CB, GL, MC with Sally Hopewell (SH), Lehana Thabane (LT) and Claire Chan (CC) invited to join the group to develop the reporting checklist using a consensus approach [10].
	2015 NIHR glossary [11]	Modified wording of descriptions for mutually exclusive pilot and feasibility studies
	Pilot and feasibility studies journal official launch (2015) [12]	BMC launched *Pilot and Feasibility Studies* journal with Gill Lancaster as Editor in Chief in 2014. The rapid growth of the journal led to Lehana Thabane joining as co Editor in Chief in 2017
2016 to date	2016 Eldridge [1]	PAFS group suggests that feasibility is the overarching concept. All studies addressing feasibility can be classified as feasibility studies but only a subset are pilot studies
	Consort extension guidance 2016 [13, 14]	CONSORT extension for pilot and feasibility studies published in *BMJ* and *Pilot and Feasibility Studies*
	March 2018 GuEST consensus workshop [15]	SE, CB, GL invited to MRC GuEST Consensus Workshop on exploratory studies
	May 2019 Internal pilot workshop [16]	Growing interest in internal pilot studies. SE, CB, SH, GL and MC invited to attend MRC Hubs for Trials Methodology workshop to discuss internal pilot studies.

In 2016, in line with our original plans, we published the reporting guideline for feasibility and pilot trials as a CONSORT extension [13, 14]. In the interim, the publication of pilot and feasibility studies had been facilitated by the launch of the BMC journal, Pilot and Feasibility Studies [12] which has seen a steady increase in submissions over its 6-year life. The aim of the journal is to provide a forum for discussion around this key aspect of the scientific process, and ensure that these studies are published, so as to complete the publication thread for clinical research.

Concurrently, there has also been increasing interest from methodological experts on the design of pilot and feasibility studies, and a greater value placed on them, by funding bodies, as essential building blocks in intervention trial development. This is seen as part of a growing focus on efficiency in trial development and design [17] and includes debate on the relative merits of internal and external pilot trials. Whereas an external pilot trial, as the name implies, does not use any of the data collected in the final analysis of any subsequent main trial, an internal pilot trial is an integral first part of the definitive trial, with internal pilot trial data contributing to the final data set.

The recent methodological debate has included discussion of the term ‘exploratory study’ [15, 18]. This term has been used to describe a small underpowered study designed and written up in a similar way to a main trial with inappropriate emphasis on *p*-values and has been used to describe the sort of studies to which our framework ascribes the terms feasibility and pilot. In 2018, this term was debated alongside the terms ‘pilot’ and ‘feasibility’ studies at the consensus workshop run by the GUEST (GUidance for Exploratory STudies of complex public health interventions) group whose research was funded by the MRC. The term ‘exploratory study’ was subsequently not included in the updated MRC recommendations for complex interventions [19, 20]. We have therefore not referred to it specifically in the remainder of this article.

The aim of this article is to provide an update on our previously published framework that incorporates the current debates taking place in the pilot and feasibility landscape. The objectives are to incorporate three particular aspects of the current debate: (i) how aspects of uncertainty about feasibility investigators are aiming to understand and influence design decisions; (ii) the interdependency of theoretically informed pilot and feasibility study aims, the external funding environment and the policy agenda; and (iii) how internal pilot studies fit into the framework.

## Expanding the 2016 conceptual framework for pilot and feasibly studies

The principles on which the 2016 conceptual framework were developed are summarised in this next section followed by a proposal to update it to include internal pilot studies. During development of the CONSORT extension for pilot and feasibility trials [10], we went through a systematic consensus exercise to agree definitions which encompassed any feasibility or pilot work. A fundamental principle was that extrapolating from standard dictionary definitions [1], we accepted that feasibility is an overarching concept to explore aspects of an intervention or of trial design for which more information was required before progressing. We termed this need for more information the ‘uncertainty’ (see Table 2).

**Table 2:** Definitions for pilot and feasibility studies as articulated in our conceptual framework [1]

Feasibility study as defined in our framework:	
Feasibility is a concept encapsulating ideas about whether something will work. A feasibility study asks whether something can be done, should we proceed with it, and if so, how?	
Pilot study as defined in our framework:	
A pilot study is a study in which a future study or part of a future study, is conducted on a smaller scale to ask the question whether something can be done, should we proceed with it, and if so, how?	
Corollary: all pilot studies are feasibility studies but not all feasibility studies are pilot studies	

We concluded that, at an early stage of developing an intervention where there is maximum uncertainty, a range of different methodological approaches could be used appropriate to the specific nature of the uncertainty. This mirrored the approach advocated in the MRC Framework for developing and evaluating a complex intervention at that time [8]. A feasibility study could be a qualitative exploration of stakeholders’ views on the acceptability of or specification for a proposed new service or an epidemiological study confirming or refuting the need for the proposed service [21]. Examples of non-randomised feasibility studies are given in Lancaster and Thabane’s editorial [22]. Systematic reviews of subject specific topics can also be an important methodology to include at this early stage [23]. Where there is less uncertainty, a pilot study might be conducted in which all or part of the proposed intervention or other process to be undertaken as part of a definitive trial are evaluated [24]. As uncertainties are resolved, a non-randomised before and after study might be conducted for the intervention arm only [25]. When most uncertainty has been resolved, a randomised pilot trial (external or internal to the main trial) might be more appropriate. An external pilot is likely to include either all or part of the definitive RCT, but on a smaller scale; by definition, it includes the randomisation process [26], but it might explore alternative recruitment and randomisation approaches. This often represents the end of the feasibility pathway, but the pathway is not necessarily, and indeed often is not, linear, a fact acknowledged explicitly in our framework.

**Fig. 1:**
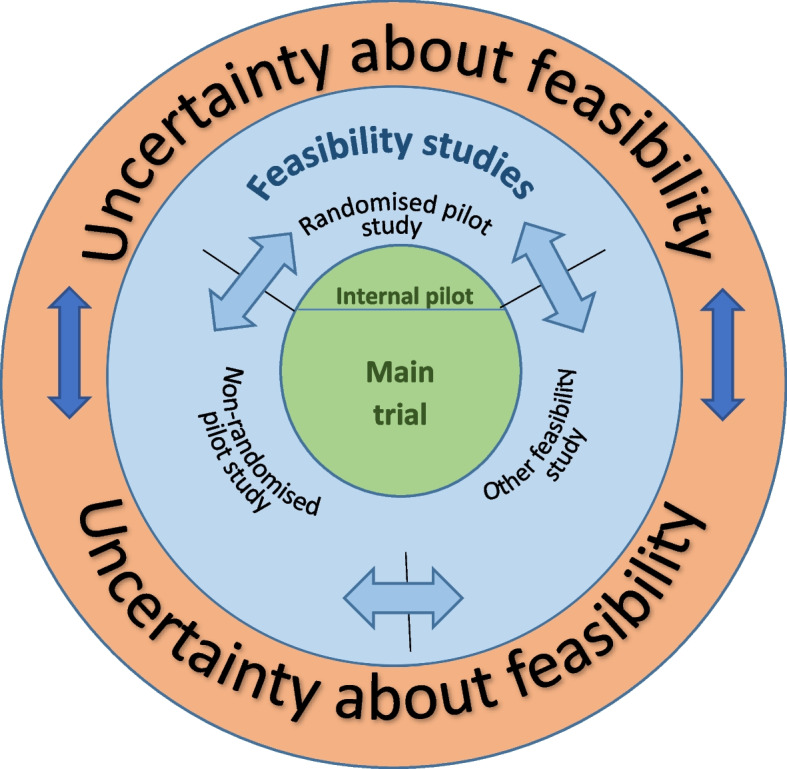
Conceptual diagram illustrating relationship between different types of feasibility studies, adapted from PLOS One

The framework (shown below in Fig. 1) includes in the innermost circle the main or definitive trial, and the three categories of external feasibility studies (randomised pilot, non-randomised pilot and other feasibility) are represented in the blue concentric circle. The two-directional arrows underpin the basic principle that the process of confirming feasibility is iterative because, paraphrasing the words of a famous American, as well as known unknowns there are unknown unknowns [27]. These unknown unknowns may only emerge once preliminary empirical work has been undertaken and may require any of the other feasibility approaches to resolve, sometimes challenging earlier planned timelines.

### Comparing internal and external pilot studies

In our original model, we focussed on feasibility studies that are external to the main definitive study, represented by the innermost green circle. Whilst for completeness we did include internal pilot studies in our diagrammatic model, we did not consider them any further. However, as noted in the introduction, there is an increasing trend to incorporate an internal pilot study within the main definitive trial. Usually, such internal pilot studies are conducted when most feasibility issues have been resolved, and the remaining uncertainties are generally focussed only on recruitment and randomisation. Indeed, the authors of a recently published systematic review of publicly funded trials have recommended that internal pilot studies should be used when evidence is needed for estimating recruitment, randomisation and attrition rates [28]. It is likely that this point would be reached only after preliminary feasibility work, which may have included an external pilot trial. It is important to note, however, that there is no black and white rule for when an external or internal pilot study should be used; both have their place.

As mentioned above, an internal pilot may be the preferred option if the only remaining uncertainties are about recruitment randomisation and attrition. Indeed, whether or not investigators undertake a formal internal pilot, it would be unusual for these aspects not to be monitored (and sometimes lead to modifications) in a definitive trial. However, if other uncertainties remain, particularly if they are considerable, an external pilot may be called for. Recruitment, randomisation and attrition could also be explored within an external pilot. However, the rates achieved in a pilot study may not always be replicated in a subsequent definitive trial as illustrated in a study of pharmacist prescribing in nursing homes. Building on a successful non-randomised pilot study [25], recruitment of sites to the study was much more challenging in the main definitive trial (unpublished data).

If the proposed definitive trial includes a novel aspect, this is likely to be a strong reason for undertaking an external pilot study, for example, if a new unit of randomisation is being proposed, or, a new trial design, or a new clinical setting. As a result of one of the pilot studies in Table 3 (FEMUR [29]), the definitive trial was deemed not feasible, and for one of the studies (UK-BEAM [30]), there was a decision to change the main trial design by abandoning practice level randomisation. Such re-design would have been difficult to implement following an internal pilot. In general, if a study is recruiting in a new context, or in an under-researched speciality or vulnerable or under-researched group, or uses an innovative design, then feasibility might be explored best in an external pilot trial. Conversely, whilst the COMQUOL [31] pilot study showed that a definitive trial was feasible, the subsequent trial was never undertaken because funding could not be secured. Nevertheless, it provides evidence for the feasibility of trials in secure mental health wards. It is an example of how well-conducted external pilot and feasibility study can provide valuable information to the wider research community, even if not of direct value to the researchers themselves. Finally, the STarT MSK [32] trial started as an internal pilot study but became de facto an external pilot when extensive changes had to be made.

**Table 3:** Examples of useful external pilot studies

Study	Feasibility uncertainty	Outcome
FEMUR study [29] ^+^	Testing if it would be possible to **randomise by primary care groups** (in the 1990s) as a precursor to a trial to see if a whole systems approach could reduce falls in older people	Showed that it was unlikely to be possible to achieve an effect given the intervention proposed within primary care groups and the main trial did not proceed
UK BEAM [30]	Testing a cluster randomisation design with back pain patients recruited from general practices (the clusters) after randomisation	Changes needed to be made in the main trial design (moving from cluster randomisation to individual randomisation) prior to proceeding to the definitive trial because of a phenomenon in cluster randomised trials now well-known: identification and recruitment bias
COMQUOL [31]	Testing feasibility of randomising **secure mental health wards** (no large definitive trials in this area)	Confirmed feasibility of this approach and funding was sought for main trial, but was never secured
STarT MSK [32]	Testing recruitment rate and GP fidelity to an in intervention testing a tool to manage musculoskeletal pain	Neither recruitment rate nor GP fidelity were met, but retention was good and when the tool was used treatment was mostly in line with the tool. Based on GP feedback, the trial processes were modified prior to proceeding to the main trial

## Implications for funders, policy makers and researchers

As trial methodology has evolved, the importance of recruiting to target, underpinned by a justified power calculation has become paramount. McDonald et al. [33] reported that in a cohort of 114 trials funded by UKMRC and HTA between 1994 and 2002, less than a third achieved their original recruitment target and half were awarded an extension. Disappointingly, despite methodological developments, little improvement was reported for studies funded between 2004 and 2016 [34]; only 50% achieved the original target recruitment and a third extended their recruitment. Whilst in some cases trials have been continued with revised recruitment targets, other trials have been closed down prematurely by funders when it became clear during the trial that target sample sizes would not be achieved. It is not surprising, therefore, that conducting extensive feasibility work prior to a full trial has more recently become a prerequisite of securing substantive research funding from most grant giving bodies. The average budget for a full trial, based on recently funded NIHR trials, has been estimated as just under £1.2m (range £321,403–£2,099,813) [17]. It is therefore in the funder’s interest to only award grants to those trials with evidence from feasibility studies that they are likely to succeed, in other words that they have demonstrated they can recruit and retain participants and implement the intervention successfully. The issue of waste in research at all stages of the research pathway was highlighted some years ago in a seminal Lancet series of five papers published in 2014, one of which focussed on the importance of considering what sort of research to fund [35] and another on more efficient regulation and funding of research [36]. However, despite this, there is little evidence that funders in general consider these issues in their decision making [37]. The NIHR is cited as a funding body that does have a more transparent approach to funding decisions; a recent study examining outcomes of feasibility studies funded through their Research for Patient Benefit schemes concluded that these studies can potentially avoid waste and ‘de-risk funding investments of more expensive full trials’ [17].

### Implications of extensive preliminary work

The consequence of the need for extensive preliminary work is an extended timeline from conceptualisation of an idea to completion of the definitive randomised controlled trial. This is a major challenge for funders and researchers and this increased time delay introduces a different sort of waste. It is estimated that the total time from initial work to completion of the definitive RCT is about 8 years [17]. Indeed, even after a successful pilot, the main trial may never happen if a further grant application needs to be written and funding secured; in the interim, priorities can change and different selection panels or funders may be involved. Further, policy makers needing evidence to inform service redesign or clinical decision making have the dilemma of delaying their decision or making a decision in the absence of the best evidence and/or commissioning a post implementation evaluation. Some short circuiting of the developmental process is therefore desirable and sometimes can be achieved by securing programme funding, or equivalent, as is possible in some countries such as the UK [38], USA [39], and Canada [40]. This longer-term award can enable seamless progression through a series of studies along the feasibility pathway culminating in a definitive trial with embedded progression criteria and stopping points. However, securing such large programme grants is challenging, often requires a two or three staged application process, and in practice is only likely to be awarded to senior investigators with a strong track record. It is not an option for less experienced research teams, and all researchers whatever their level of experience are constrained by competitive funding systems and limited funding budgets.

**Fig. 2:**
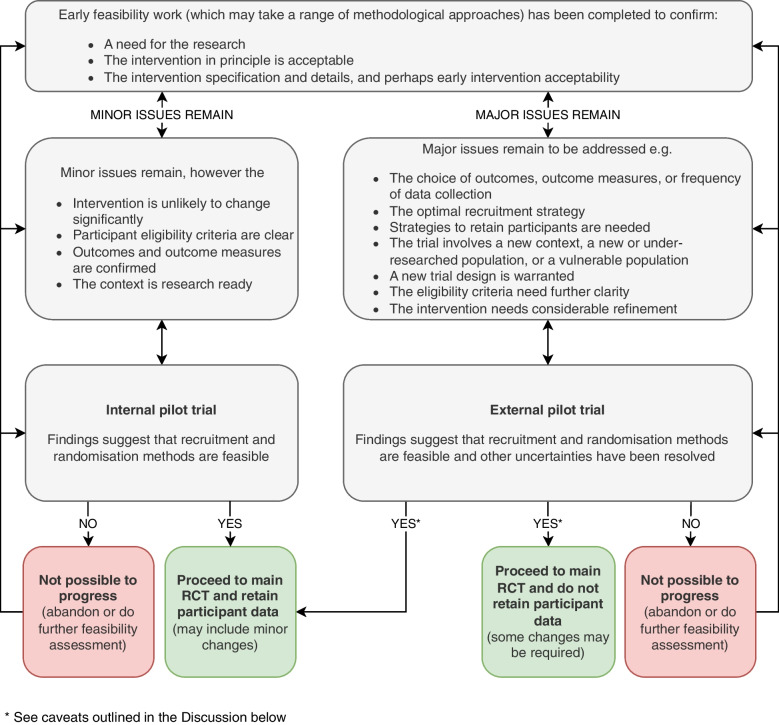
Simplified flow chart incorporating both internal and external pilot studies

Ultimately, pragmatism may often over-ride methodological reasoning and scientific principles. Study design and approaches to securing funding may relate to seniority of research leads, as well as being based on how to explore specific uncertainties. Criteria for successful funding often include a request for reviewers to comment on the track record of the applicants, especially the lead applicant. A junior researcher seeking a first opportunity to become a principle investigator would not apply for a programme grant but might reasonably be able to secure funding for a smaller pilot study, designed as part of a programme of work leading to a definitive trial. Indeed, many funding bodies with limited budgets explicitly prioritise pump priming projects such as pilot studies, including external pilot trials. Whilst these may not be definitive in their own right, they have the potential to seed bigger subsequent grants. If money is available immediately for a definitive trial, perhaps through a commissioned call, then the balance may swing in favour of an internal pilot, with the benefit of reducing the timeline and allowing for some ongoing uncertainties to be resolved as the main trial proceeds. But there are times when care needs to be taken. Whilst challenges in recruitment can often be compensated for by extending to other centres, other criteria, for example based on safety, which might require further training of the service providers, could necessitate a fundamental change in the intervention and could invalidate inclusion of the internal pilot participants. The risk of such events needs to be recognised. The flow chart below (Fig. 2) illustrates in simplified form the options for study design. Given the preceding discussion, it is acknowledged that at all points in the decision-making process the options for funding may also be a consideration. As the flow chart suggests, internal and external pilot trials are not a dichotomy. There is considerable overlap in their objectives and the uncertainties they address, reinforcing that choice is often pragmatic as mentioned above and not always based on methodological requirements

## Discussion

It is noted that many of the approximately 3000 papers citing our previous work [1, 13, 14] do so as justification for doing a feasibility study. If we have achieved a paradigm shift in research culture, then this suggests one of the aims we originally had for undertaking this work has been achieved. Publication has also been facilitated by the launch of the Pilot and Feasibility Studies Journal, dedicated to publishing early developmental work but emphasising that this must be true feasibility work not a small underpowered, non-generalizable study. However, disappointingly, we know that the latter do still exist.

There is now popular consensus that early work to identify the optimal approach to recruiting and confirming the size of the eligible population is paramount. Additionally, the increasing introduction of complex interventions in service delivery has driven the need to understand the contents of the intervention—the black box—and to ensure as far as possible that all the components are delivered in an optimum way to maximise the chance of a successful trial outcome. In other words, it is important to ensure that a good idea is not rejected because of a failure of one part of the system which should have been identified and addressed before progressing to a definitive study. All of the above increasingly validate the systematic development approach described in all versions of the MRC Framework for developing and evaluating a complex intervention [18]. Now in its fourth version, its focus is on identifying what is uncertain and conducting work to remove that uncertainty. All versions of the MRC framework have included details on the need for feasibility work, although terminology and emphasis has subtly changed. Our conceptual framework sits well within this paradigm.

We have made the case that both external and internal pilot trials are part of an armamentarium of research approaches that can be used to address uncertainty. There is no absolute rule about when either should be adopted, and we have emphasised the importance of balancing the ideal of eliminating all uncertainties against the pragmatic need for efficiency and value for money. With that in mind, a further area of enquiry might be whether an external pilot trial can become an internal trial if nothing has been changed as shown in the flow chart in Fig. 2. The implications of this would be that data collected as part of the external pilot trial would be included in the definitive trial data set. Issues that then need to be considered could include whether there have been any external contextual changes in the time lapse between completion of the pilot trial and start of the definitive trial, and the effect these might have preferentially on either arm. All of these options would need to be prespecified in the protocol and in the interests of transparency also included in other documentation such as participant information and consent. In the longer term, clear guidance on all of this is required.

There are other aspects of the optimal design and conduct of feasibility studies, which have not been discussed in this paper but are mentioned here as examples of further work required to inform methodological guidance. In line with the CONSORT extension, studies should have in place progression criteria (Checklist item 22a), yet recent work by our group suggests just under a fifth of pilot study protocols include progression criteria [41]. Progression criteria should ideally be facilitative and used to inform successful trial completion, not as a tool to stop a trial. These may be particularly challenging for an internal pilot trial where the potential for further change is limited. If recruitment is slower than expected, what is the extent of change that can be made whilst retaining the internal pilot data? If intervention fidelity is poor, can any changes be made, for example more training provided, and differences accounted for in analyses? If recruitment is slow can eligibility criteria be relaxed, the timeline extended or the number of sites increased? Can secondary outcomes be changed or reduced or data collected differently or at a different time interval in order to improve response, as long as the primary outcome is unchanged?

There is also a lot of current debate on the correct basis for determining the pilot study sample size with consensus that there will rarely be a single right answer. However, whatever sample size is chosen, and how it is chosen, balancing all the competing factors, it must be scientifically justified. For if the study is not designed with an appropriate sample size, it is unlikely that its findings will be valid. Lewis et al. have begun to address sample size for process outcomes to inform progression criteria in pilot and feasibility studies [42].

Finally, there is the ethical question of the conditions of the participant’s informed consent. In the strictest terms of transparency under GDPR, participants consenting to take part in a randomised pilot trial should be clearly informed how their data will be used. A review of 184 studies submitted to a Canadian Research Ethics Committee suggests the transparency of informed consent in PAFS is inadequate and needs to be specifically addressed by research ethics guidelines [43]. This is not just about having the words pilot or feasibility in the title. For an external pilot trial, it should be explicit that the findings of the study will only be used as part of the research development process and not part of a data set used to provide definitive evidence. The implications of this transparency, however, have not yet been fully debated. For, if the purpose of the pilot trial is to (say) assess recruitment rates, how valid is the pilot with respect to the main trial if people are told there is a different purpose to the research? In the interests of research and public good is it justified to deceive? For participants recruited to an internal pilot trial, what should they be told? Our early work suggests just under a fifth of studies declare their pilot or feasibility objectives in the Participant Information Sheet [43]. Changing an external pilot to an internal pilot—one of the options we speculate on above—might also have implication for informed participant consent, GDPR and the way the data is used. This is another area requiring exploration.

## Conclusion

In conclusion, in this paper, we have revisited our rationale requiring that pilot and feasibility studies should be clearly defined and recognised as study designs in their own right. We have now fully integrated internal and external pilot studies into our model and considered their continuous as opposed to dichotomous relationship. In this final discussion, we have raised awareness of issues common to both internal and external studies which are in themselves current uncertainties in the context of optimum research design. Resolving all of these would contribute to the delivery of research which is more ethical, rigorous, efficient and above all robust in its findings.

## Data Availability

Data sharing is not applicable to this article as no datasets were generated or analysed during the current study.
